# Improved Functional Assessment of Ischemic Severity Using 3D Printed Models

**DOI:** 10.3389/fcvm.2022.909680

**Published:** 2022-06-30

**Authors:** Kranthi K. Kolli, Sun-Joo Jang, Abdul Zahid, Alexandre Caprio, Seyedhamidreza Alaie, Amir Ali Amiri Moghadam, Patricia Xu, Robert Shepherd, Bobak Mosadegh, Simon Dunham

**Affiliations:** ^1^Department of Radiology, Dalio Institute of Cardiovascular Imaging, Weill Cornell Medical College, New York, NY, United States; ^2^Department of Mechanical and Aerospace Engineering, Cornell University, Ithaca, NY, United States

**Keywords:** CCTA, radiology, 3D printing, *in vitro*, blood analog fluid, fractional flow reserve, catheterization

## Abstract

**Objective:**

To develop a novel *in vitro* method for evaluating coronary artery ischemia using a combination of non-invasive coronary CT angiograms (CCTA) and 3D printing (FFR_3D_).

**Methods:**

Twenty eight patients with varying degrees of coronary artery disease who underwent non-invasive CCTA scans and invasive fractional flow reserve (FFR) of their epicardial coronary arteries were included in this study. Coronary arteries were segmented and reconstructed from CCTA scans using Mimics (Materialize). The segmented models were then 3D printed using a Carbon M1 3D printer with urethane methacrylate (UMA) family of rigid resins. Physiological coronary circulation was modeled *in vitro* as flow-dependent stenosis resistance in series with variable downstream resistance. A range of physiological flow rates (Q) were applied using a peristaltic steady flow pump and titrated with a flow sensor. The pressure drop (ΔP) and the pressure ratio (P_d_/P_a_) were assessed for patient-specific aortic pressure (P_a_) and differing flow rates (Q) to evaluate FFR_3D_ using the 3D printed model.

**Results:**

There was a good positive correlation (*r* = 0.87, *p* < 0.0001) between FFR_3D_ and invasive FFR. Bland-Altman analysis revealed a good concordance between the FFR_3D_ and invasive FFR values with a mean bias of 0.02 (limits of agreement: −0.14 to 0.18; *p* = 0.2).

**Conclusions:**

3D printed patient-specific models can be used in a non-invasive *in vitro* environment to quantify coronary artery ischemia with good correlation and concordance to that of invasive FFR.

## Introduction

Obstructive coronary artery disease (CAD) is one of the most common type of cardiovascular disease (1). The evaluation and diagnosis of CAD remains a challenging task. Anatomical and functional assessment through invasive coronary angiography (ICA) is the current reference standard to indicate the presence, location, and extent of a stenosis/obstruction. Stenoses that are functionally significant (flow limiting/ischemia causing) need to be treated invasively to reduce CAD morbidity (2–5). On the contrary, invasive treatment of functionally non-significant stenoses may lead to harmful outcomes (3, 6). Thus, the independent evaluation of this disease either by non-invasive or invasive approaches is of utmost importance for the selection of appropriate and optimized therapeutic methods such as bypass surgery, stents or drug therapy while treating a patient.

Non-invasive imaging methods like coronary CT angiography (CCTA) not only help identify patients with suspected CAD, but also allow for visualization/quantification of the coronary artery stenosis (7). Although CCTA has high sensitivity in determining the functional significance of the stenosis and ruling out CAD, its corresponding specificity is lower (8–11). Hence, patients with obstructive CAD typically undergo an additional procedure like ICA to further determine the functional significance of the stenosis by invasively measuring the fractional flow reserve (FFR; ratio of average pressures distal [P_d_] and proximal [P_a_] to a stenosis at maximal hyperemia). FFR is the current clinical gold standard for both establishing the functional significance of a stenosis and also to guide its treatment. In order to reduce the number of unnecessary invasive procedures, non-invasive determination of the functional significance of stenoses based on CCTA images is being extensively investigated (12–14).

Recently, 3D printing, an additive manufacturing technique that enables direct fabrication of physical models based on digital objects of arbitrary geometry, has become more common; particularly for medical device and healthcare applications (15, 16). The purpose of this research is to develop a novel *in vitro* method to evaluate coronary artery ischemia using 3D printed coronary arteries whose 3D geometric features are derived from non-invasive coronary CT angiograms (CCTA). An *in vitro* flow circulation system representative of invasive measurements in a cardiac catheterization laboratory was developed to experimentally evaluate the hemodynamic parameters of pressure and flow across patient-specific 3D printed models. Our overall goal is to develop a novel non-invasive system for determining patient-specific thresholds of ischemia and to validate this system in a unique clinical trial of individuals with comprehensive physiologic measurements. In this pilot study, the concordance between the FFR values measured *in vitro* using 3D printed models (FFR_3D_) and the gold standard (invasive FFR) is also examined.

## Methods

This study was performed as a prespecified secondary aim of the CREDENCE study, to investigate the mechanism by which plaque characteristics may impact fractional flow reserve *via* their material properties (17). An *in vitro* flow circulation system representative of invasive measurements in a cardiac catheterization laboratory was developed to experimentally evaluate the hemodynamic parameters of pressure and flow across a pilot cohort of twenty eight patient-specific 3D printed coronary artery models. Experiments were based on patient's image data and hemodynamic parameters, which were De-identified prior to study. The details of the study population and experimental setup are discussed below.

### Study Patients

A random subset of twenty eight patients from the multicenter CREDENCE trial (Clinical Trials Gov., ID: NCT02173275) were included in this pilot study. The CREDENCE trial is a prospective, multicenter diagnostic derivation-validation controlled clinical trial that recruited 612 stable patients, without a prior diagnosis of CAD from 2014–2017. Patients were recruited across 17 centers in the Unites States, Netherlands, Japan, China, Latvia, Italy, and South Korea. The rationale and design of the CREDENCE trial has been described in a previous study (17). Briefly, enrolled patients underwent both CCTA and Myocardial perfusion imaging tests (MPI), followed by invasive coronary angiography (ICA) with FFR measurements in three epicardial coronary arteries. Eligibility criteria included referral to non-emergent ICA according to the American College of Cardiology/American Heart Association clinical practice guidelines for stable ischemic heart disease (18, 19). All the non-invasive and invasive imaging tests were interpreted blindly by core laboratories. The institutional review board of each enrolling site approved the study protocol and all patients provided written informed consent.

### Image Acquisition

CCTA imaging was performed using a single or dual source CT scanner with at least 64-detector rows and a detector row width of ≤0.75 mm (17). Scans were performed retrospectively (65%), prospectively (27%), or with single-beat (8%) acquisitions. Sites were instructed to perform CCTA in accordance with quality standards set forth by the Society of Cardiovascular Computed Tomography (SCCT) guidelines (20). The CCTA images were exported into a DICOM (Digital Imaging and Communications in Medicine) format. Patient-specific 3D coronary artery models were then segmented from this CT volume data, in DICOM format, using Mimics image processing software (Mimics 18.0, Materialize, Leuven, Belgium).

### Image Segmentation and 3D Printing of Coronary Vessel Models

**Figure 1:**
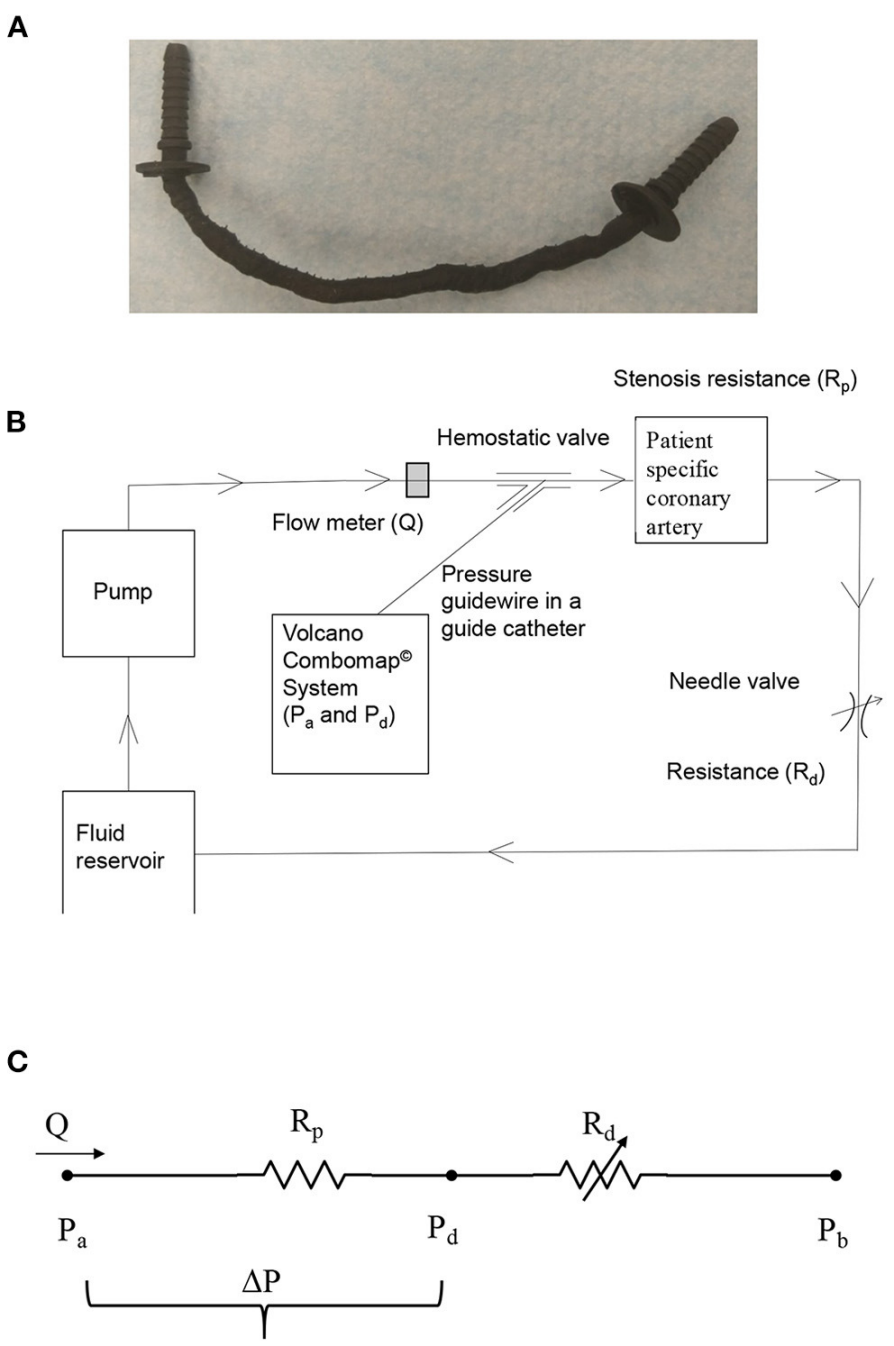
**(A)** 3D printed patient-specific LAD coronary artery; **(B)** schematic of *in vitro* coronary flow-loop setup for FFR_3D_; and **(C)** electrical analog model of the flow-loop with two resistances in series. R_p_, stenosis resistance from the 3D printed model; R_d_, coronary microvascular resistance; Q, flow through stenosed artery measured by the flowmeter; P_a_, aortic pressure; P_d_, pressure distal to the stenosis; ΔP = P_a_-P_d_, pressure-drop across stenosis; P_b_, coronary outflow pressure.

In total, twenty eight patient-specific 3D coronary artery models [five right coronary artery (RCA), five left circumflex coronary artery (LCX) and 18 left anterior descending artery (LAD)] have been segmented from the CCTA images of 28 patients. Segmentation was performed by defining a range of thresholding value to obtain the segmentation mask of the region of interest (blood volumes for the aorta and coronary vessels) in Mimics (Mimics 18.0, Materialize, Leuven, Belgium). The thresholding value for the region of interest in general different for different patients but is within the range of soft tissue. After setting an optimal thresholding value, a region growing function was used to generate the coronary artery mask along with some unwanted mask (mask volumes unrelated to coronaries) which is later edited manually. This segmented mask was then assessed by an independent experienced cardiologist (SJ) who is blinded to both CCTA and ICA results. A patient-specific 3D aorto coronary lumen surface model, from this final segmented mask, was saved in the Steriolithography (STL) geometric file format. The meshes were then moved to Geomagic^TM^ to simplify the mesh (by linear subdivision) and add smoothness to the mesh. The corresponding coronary vessel of interest (RCA/LCX and/or LAD) without branches, from this aorto-coronary surface geometry (lumen), was then: (i) extracted; (ii) thickened outwards by 1 mm from the lumen surface to represent arterial thickness; and (iii) coupled with appropriate barb fittings at its inlet and outlet in solid works (Dassault Systemes, France; Figure 1A). These coronary vessel models with barb fittings in STL geometric format were then printed using a Carbon M1 3D printer (Carbon Inc., California, United States) with urethane methacrylate (UMA) family of rigid resins. The Carbon M1 3D printer uses an additive manufacturing methodology called projection stereolithography apparatus (21) (SLA); which builds/fabricates models layer by layer using a curable photopolymer (liquid resin). Apart from the higher resolution, an additional advantage of using the SLA methodology to 3D print the coronary vessels is that there is no support material in the lumen that needs to be removed post-printing [for e.g., like in PolyJet printing method (22)]. Thus, 3D printing the models using an SLA methodology not only saves time (post-print cleaning), but also yields a smooth lumen surface in the 3D printed model, similar to that in the 3D geometric model.

### *In vitro* Flow Circulation System

The coronary flow system, shown in Figure 1B, was used to perform the *in vitro* experiments under physiologic steady flow conditions of pressure and flow. The flow was maintained to be quasi-steady in the flow system and mean flow rate was used as the relevant maximum flow rate scale (perceived hyperemia). A 60:40 mixture (by volume) of distilled water and glycerol (Shelley Medical Imaging Technologies, Ontario), having a viscosity (4.5 cP) and density (1.04 g/cm^3^) similar to that of blood was selected for use in the experiment as the Newtonian blood-analog fluid (BAF). Previous studies have shown that Newtonian assumption has lesser influence on flow field in medium to large sized arteries such as coronary artery (23–25).

A Cole-Parmer digital gear-drive pump (model # EW-74014-42) was used to impart and vary the flow rates in the flow system. The circulation system was modeled as a flow-dependent stenosis resistance (R_p_) in series with an adjustable downstream resistance (R_d_; needle valve [model # EW-06394-04]) (26). The corresponding electrical analog of the model is shown in Figure 1C. The fluid reservoir is open to atmosphere, thus assuming P_b_ in Figure 1C to be zero. The fluid reservoir, pump, 3D printed coronary vessel model and the needle valve were connected to form a closed loop using flexible Platinum-cured Silicone tubing.

### Experimental Setup

In order to mimic the pressure measurement in a cardiac catheterization laboratory setting, a 5F diagnostic catheter was advanced proximal to the stenosis section through a cannula. The aortic pressure (P_a_) was measured through a fluid-filled line connected to a Namic disposable transducer (Navilyst Medical) and the coronary guiding catheter. “A 0.014” pressure sensor-tipped guidewire, connected to a Volcano ComboMap machine (Volcano Corp.,), was set to zero, advanced *via* an introducer needle and a hemostatic valve through the diagnostic catheter. The pressure sensor-tipped guidewire was then calibrated, normalized to the diagnostic catheter, and advanced distal to the stenosis section. The pressure distal to the stenosis (P_d_) was measured through this pressure sensor-tipped guidewire. Inlet flow rate into the stenosis test section was measured using a transit-time ultrasound clamp-on flow sensor (Transonic Inc.,TS410-ME4PXL).

### Experimental Protocol

The 3D printed patient specific coronary vessel models were fixed in the flow system one at a time, as shown in Figure 1B. The BAF was then allowed to circulate through the flow system for about 5 min prior to the experiment in order to achieve steady state conditions and care was taken so that the flow loop did not have any air-bubbles during the experiment. The aortic pressure (P_a_) for each 3D printed model was maintained at a constant value as measured during invasive coronary angiography for the corresponding patient. This constant inlet aortic pressure (P_a_) condition is achieved, under different flow rates, by varying the needle valve resistance (R_d_) that mimicked adjustable microcirculatory resistance. The distal pressure (P_d_) for each varying flow rate was measured only after pulling back the pressure guidewire into the diagnostic catheter, renormalizing and advancing across the stenosis, to avoid the effect of drift on the measurements. Three sets (*n* = 3) of experiments were carried out and the three pressure-flow data sets were averaged to obtain the pressure drop- flow rate (ΔP–Q; Supplementary Figure 1) for each. The pressure ratio (P_d_/P_a_) at differing prescribed flow rates (Q), applied to each of the 3D printed model was then assessed from the ΔP–Q curves (Supplementary Figure 2).

### Determination of Hyperemic Flow

The physiological flow conditions like pharmacologically induced hyperemia are unknown in the *in vitro* experimental setup. However, the methodology for estimating *hyperemia* using maximal vasodilation-distal perfusion pressure plot (CFR-P_d_) was previously proposed by Kirkeeide et al. (27) and reported in an *in vitro* setting by Sinha Roy et al. (28) assuming a resting blood flow rate of 50 mL/min for a 3 mm native diameter vessel. Utilizing this resting blood flow value, the hyperemic flow rates, Q_h_, were obtained using the *intersection* of the (CFR-P_d_) line and the experimental ΔP–Q curve (Supplementary Figure 3). The CFR-P_d_ line was a linear curve fit based on previously reported clinical data from 32 patients' (29) group with normal microvasculature. These patients had no evidence of myocardial infarction (MI), no left ventricular hypertrophy, no valvular heart disease, and a normal left ventricular ejection fraction. The *Y*-intercept of the CFR-P_d_ line is denoted as zero-flow mean pressure (P_zf_), which represents the residual pressure at no flow. Physiologically realistic P_zf_ values of 20 mmHg, as reported in a previous clinical study (30, 31), were also used in the maximal vasodilation CFR-P_d_ line (Supplementary Figure 3). The distal bed-resistance (R_d_) offered by the microvasculature to the flow can then be evaluated as below:


(1) $$\begin{array}{r} R_{d}=\frac{\left(P_{d}-\;P_{zf}\right)}{Q_{h}} \end{array}$$

### Statistical Analysis

The association between FFR_3D_ and FFR was assessed by Bland–Altman plots with 95% limits of agreement and spearman correlation coefficient. The receiver operating characteristic (ROC) curve with a corresponding area under the ROC curve (AUC) was performed to assess the per-vessel discrimination of functional ischemia by FFR_3D_, using invasive FFR ≤ 0.8 as the reference standard. Youden's index was used to determine the optimal threshold value of FFR_3D_. Statistical analyses were performed using Medcalc (Ostende, Belgium) with *p*-value <0.05 considered to indicate a statistically significant result.

## Results

### Baseline Patient Characteristics

The baseline clinical characteristics of the twenty eight patients/vessels (5 RCA, 5 LCX, and 18 LAD) are summarized in Table 1. The average age of the study population was at 65.3 ± 8.3 years. The prevalence of known cardiovascular risk factors among this cohort was 25% for diabetes, 64% for hypertension, 43% for Dyslipidemia, 36% for family history of CAD and 21% for past history of smoking. The study population consisted of intermediate coronary stenosis with a mean diameter stenosis of 53.7 ± 17.1% after quantitative CT measurements. Significant stenosis (>50 diameter stenosis) was observed in 61% of study population.

**Table 1:** Baseline patient characteristics.

**Characteristic**	**Data**
Age (y)^*^	65.3 ± 8.3
Male-to-female ratio	21:7
Diabetes	7 (25%)
Hypertension	18 (64%)
Dyslipidemia	12 (43%)
Family history of CAD	10 (36%)
Past history of smoking	6 (21%)
>50% stenosis by CT	17 (61%)
Number of vessels (LAD/LCX/RCA)	18/5/5

* *Data are means ± standard deviation*.

### Correlation and Concordance Between FFR_3D_ (*in vitro*) and FFR (Invasive)

**Figure 2:**
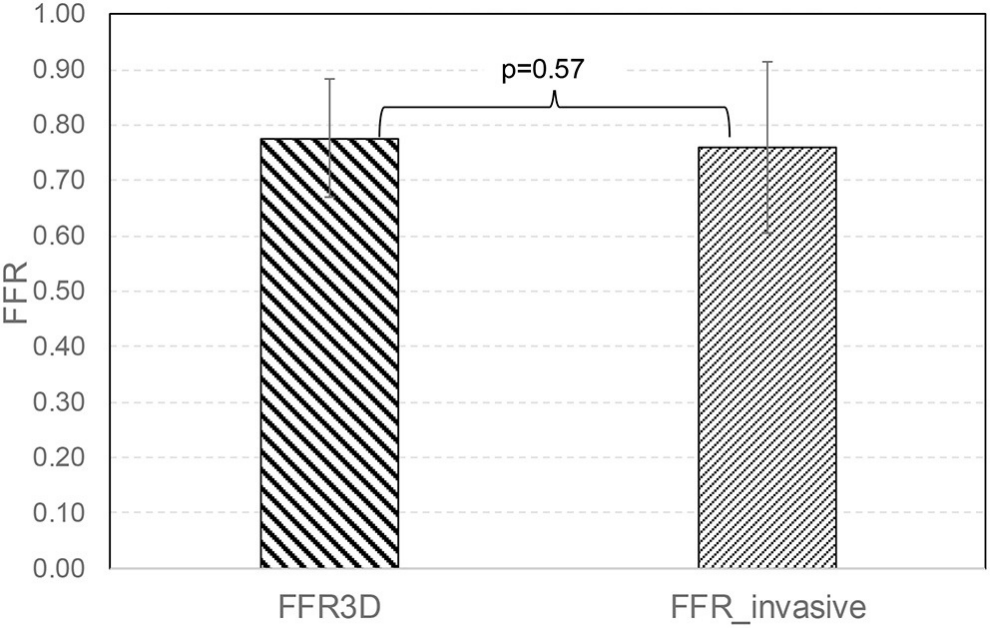
Mean value comparison between FFR_3D_ and invasive FFR.

**Figure 3:**
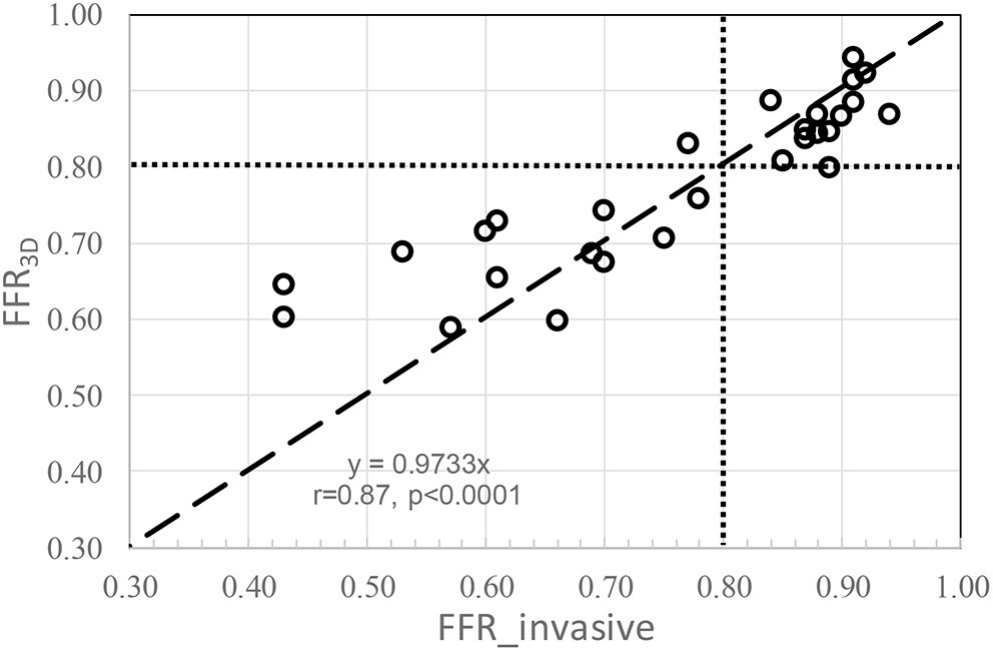
Correlation between FFR_3D_ and invasive FFR (dotted lines represent an FFR value of 0.80).

**Figure 4:**
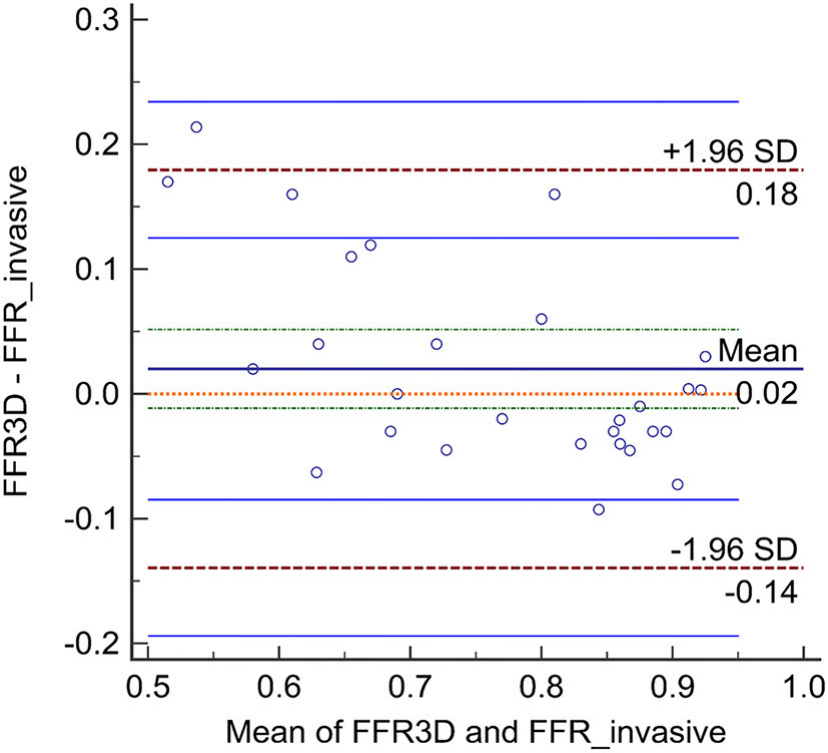
Bland-Altman analysis between FFR_3D_ and invasive FFR.

No significant difference was observed in the mean values between FFR3D and invasive FFR values (0.78 ± 0.11 and 0.76 ± 0.15, *p*=0.57; Figure 2). For these twenty eight models, there was also a good positive correlation as determined by the Spearman coefficient of correlation (*r* = 0.87, *p* < 0.0001; Figure 3) between the FFR_3D_ and invasive FFR. Further, Bland–Altman analysis (Figure 4) revealed a mean bias of 0.02 (limits of agreement: −0.14 to 0.18; *p* = 0.2), which was proportional to the average of FFR (Spearman *r* = 0.42; *p* = 0.025). Thus, suggesting that the concordance between FFR_3D_ and invasive FFR for the assessment of ischemic severity was good.

**Figure 5:**
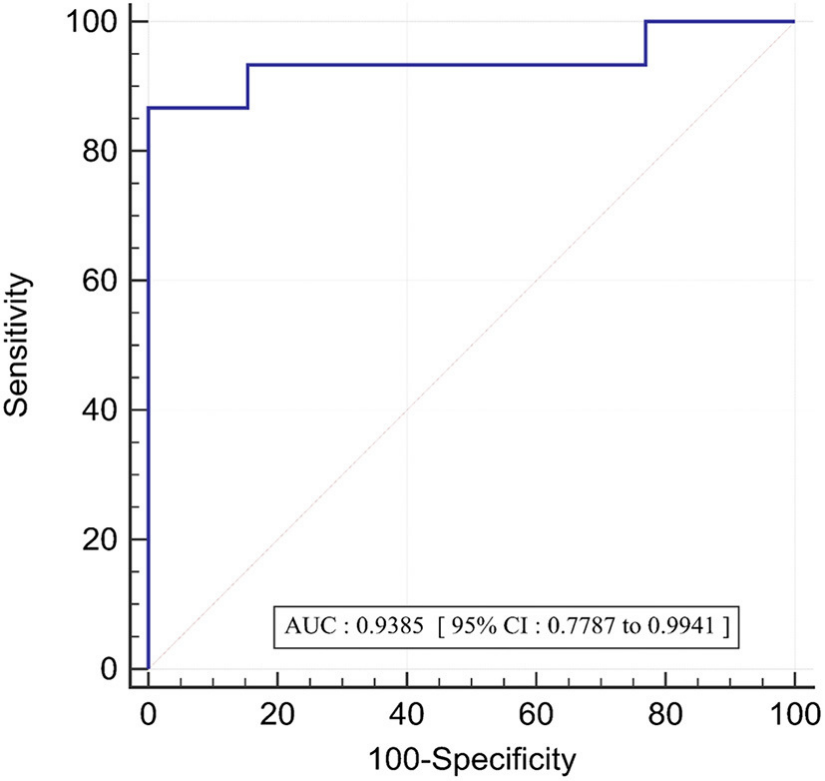
ROC curve for the prediction of functionally significant stenoses (FFR<=0.80) by FFR_3D_; AUC, area under the curve; ROC, receiver operating curve.

In the twenty eight vessels, there were 15 (54%) vessels with an invasive FFR ≤ 0.8 signifying the presence of ischemia. The area under the receiver operating curve with regards to discriminating ischemic lesions by FFR_3D_ at the invasive FFR threshold of 0.80 is displayed in Figure 5. ROC analysis for FFR_3D_ demonstrated an AUC of 0.94 (95% CI: 0.78–0.99; *p* < 0.0001). Youden's index testing establishes a threshold of ≤0.76 for FFR_3D_, sensitivity = 86.7% (95% CI: 59.5–98.3), specificity = 100 % (95% CI: 75.3–100). This threshold value correctly classified 100% of vessels.

## Discussion

An *in vitro* experimental flow-loop was developed to model physiological coronary circulation in CCTA-derived *patient-specific 3D printed* coronary vessel geometry as a flow-dependent stenosis resistance (R_p_) in series with a downstream resistance (R_d_). The *in vitro* flow circulation system was representative of invasive measurements in a cardiac catheterization laboratory. In this pilot study, twenty eight CCTA-derived *patient-specific* 3D printed coronary vessels were integrated into this flow loop one at a time to estimate the FFR value *in vitro* (FFR_3D_) and compare the same with the corresponding gold standard invasive FFR. We observed that the FFR values estimated from the 3D printed models *in vitro* (FFR_3D_) correlated well (*r* = 0.87; *p* < 0.0001) with the corresponding invasive FFR values. More importantly, in this pilot study, Bland-Altman analysis revealed a good concordance between the FFR_3D_ and invasive FFR values with a mean bias of 0.02 (limits of agreement: −0.14 to 0.18).

Coronary artery disease (CAD) is one of the main causes of morbidity and mortality worldwide (1, 32, 33). Anatomical assessment through invasive coronary angiography (ICA) remains the gold standard for the diagnosis of CAD. Invasive measurement of fractional flow reserve (FFR); a physiological adjunct to functional stenosis severity when combined with the anatomical assessment from ICA was found to outperform the anatomical assessment alone for diagnosing and guiding treatment to CAD (34, 35). However, FFR is currently used to guide only about 6% of interventions performed in the United States (36) due to limiting factors like adenosine infusion, risk of complications due to invasiveness of using pressure wire in distal vessels for e.g., vessel dissection (occurs in about 0.5% of the procedures) and patient-related contraindications (34, 37, 38) (hypotension, asthma, etc.). To overcome these limitations, there has been a recent interest in developing and using less invasive techniques for assessing both anatomy and physiology. CCTA imaging is one such non-invasive imaging modality that will allow for assessing both anatomy and physiology [for e g., using 3D lumen reconstruction and computational fluid dynamics [CFD]; FFR_CT_ (12–14)].

Conversely, for the first time, in this pilot study using CCTA images, we developed and evaluated a novel *in vitro* method to assess *physiological* ischemia from CCTA-derived and *3D printed* coronary arteries. Briefly, the programmatic workflow in the proposed methodology on a per-patient basis involves the following three steps: (i) semi-automatic segmentation of lumen from CCTA scans (about ~ 40 min); (ii) 3D printing the segmented model (about ~60 min); and (iii) plugging the 3D model into the existing flow loop to simulate patient-specific physiological conditions and estimate FFR_3D_ (about ~10 min). Thus, currently the total time required to estimate FFR_3D_ from a CCTA scan is about 110 min (~2 h). With the recent developments in automatic reconstruction of coronary arteries from CCTA using deep learning technology and also the advancements in additive manufacturing technology; we believe that we can further reduce the total estimated time to about from 40 to 30 min or even lower in future.

### Comparison to FFR_CT_

Using invasive FFR as a gold standard, the novel approach of combining CT scans and computational fluid dynamics (CFD) for estimating non-invasive FFR (FFR_CT_) was first evaluated by Koo et al. (12) and Min et al. (13) in a cohort of 103 and 252 patients, respectively. Norgaard et al. (14) in a separate study also concluded that FFR_CT_ has high diagnostic performance when compared to invasive FFR and reported that mean time to computation of FFR_CT_ results was less than 4 h. It should be noted that both FFR_CT_ and FFR_3D_ use segmentation approaches as a first step to generate the lumen geometry from CCTA scans. On this patient-specific lumen geometry, FFR_CT_ uses CFD to solve the Navier-Stokes equations for fluid flow by estimating resting flow and assuming that microcirculation reacts predictably to the physiological condition of maximal hyperemia (39). FFR_3D_, however, uses the 3D printed models to generate the characteristic non-linear ΔP–Q curves while accounting for the physiological phenomenon of coronary autoregulation and then estimates patient-specific hyperemic condition from these curves in confluence with the linear CFR-P_d_ line from previous clinical measurements (26). Moreover, the measurement uncertainty (from flow sensor and pressure wire) corresponding each data point in the non-linear ΔP–Q curve (Supplementary Figure 1) was also quantified using uncertainty analysis (40, 41). The uncertainty in pressure-ratio (P_d_/P_a_) and flow-ratio (Q/Q_b_) values at each data point due to measurement errors were found to be within 1%. In addition to the above, although the shorter time required to estimate FFR_3D_ (from this pilot study) is advantageous, we believe that a future comprehensive study comparing both FFR_3D_ and FFR_CT_ is still warranted.

### Comparison to Quantitative Flow Ratio

Recently, Tu et al. (42) proposed an alternative method, Quantitative flow reserve (QFR), of calculating FFR during in-procedure angiography (43). Briefly, the methodology involves: (i) generating a 3D vessel contour from 2D quantitative coronary angiography (QCA); (ii) followed by hyperemic flow estimation from Thrombolysis in Myocardial Infarction (TIMI) frame count using empirical relations; and (iii) utilization of CFD or simplified analytical equations to estimate QFR at hyperemia [median time for QFR estimation is 5 min (43)]. Tu et al. (42), in their initial study, reported that there was a strong correlation (*r* = 0.81; *p* < 0.001) between QFR and invasive FFR with a mean difference of 0.06 (*p* = 0.054). Similar to these results, our pilot study using 3D printing methodology (FFR_3D_) also showed a strong correlation (*r* = 0.87; *p* < 0.0001) and a lower mean bias (0.02; *p* = 0.2) when compared to invasive FFR. Although there is a substantial gain in processing time for QFR (5 min) over FFR_3D_ (110 min), it should be noted that QFR is derived from: (i) 3D vessel contour derived from 2D angiograms as opposed to volumetric data used for geometry generation in FFR_3D_; and (ii) hyperemia estimation from empirical equations as opposed to the physiological scenario of accounting for auto-regulation and micro-circulatory resistance in FFR_3D_.

### Potential Clinical Implications and Future Work

As mentioned previously, physiology-guided decision making using invasive FFR in conjunction with invasive coronary angiography can improve diagnosis and treatment of CAD. However, invasive FFR is currently used to guide only about 6% of interventions performed in the United States (36). One of the main reasons for this can be either the cost of pressure wire and/or the small risk of injuring vessels during pressure wire manipulation [for e g., Side branch dissection (37)]. Alternatively, a complete non-invasive diagnostic approach of combining both anatomy and physiology using CCTA scans and 3D printing (FFR_3D_) will incur low cost and no risk of dissection.

Currently, the estimation of FFR_3D_ requires some user interaction during the three steps of segmentation, 3D printing and flow loop evaluation. Technological advancements in deep learning based segmentation approaches, 3D printing methodologies (and materials) and using pressure sensing taps on the 3D printed model for direct pressure measurement could further automate and expedite the entire workflow. Moreover, beyond the estimation of FFR_3D_ for CAD diagnosis; the 3D model could also be used for various pre-intervention planning approaches including: (i) estimating the length and type of stent needed to open the blockage; (ii) assessment of post-intervention hemodynamics after inserting a stent into the 3D model to open the vessel; and (iii) physiologically discriminate between focal and diffuse CAD by measuring the drop in pressure across a length of a vessel (i.e., pressure gradient) (44).

### Assumptions

The wall of the stenosis geometry was assumed to be rigid in the *in vitro* experiment. A rigid wall approximation when compared to a compliant wall model is expected to provide a conservative estimate (45) (limiting case) of pressure drop as seen in hyperemia. However, further *in vitro* experiments with compliant stenosis models are needed for comparison. The resting blood flow was assumed to be a constant value of 50 mL/min in this study. Previously, in a preclinical study with anesthetized dogs, Gould et al. (46) reported that progressive reduction of coronary lumen has no effect on resting blood flow until the vessel is occluded by about 80–85% of the nominal vessel diameter. More recently, Nijjer et al. (47), in a large dataset of real-world patients, that underwent simultaneous intracoronary pressure and flow measurement, also reported that *resting flow is preserved* despite increasing stenosis severity owing to compensatory reduction in resting microvascular resistance.

The study involves predominantly focal lesions, and we assume that the side branch flow likely affects diffuse lesions differently than focal lesions. Gosling et al. (48), evaluated the effect of side branch flow on non-dimensional physiological flow indices and reported that there was no significant change/effect on the non-dimensional physiological indices (pressure based). The authors report that this phenomenon could be because side branch flow likely affects diffuse lesions differently than focal lesions. However, it should also be noted that in contrast to observations from this study, Sturdy et al. (49) and Vardhan et al. (50) reported that neglecting the side branches increased estimates of wall shear stress and pressure drop.

## Limitations

Steady state average pressure and flow values were used in this *in vitro* experiment because FFR values are defined as the mean pressure ratios. Previously, Huo et al. (51), in an *in vitro* experiment compared pressure drop between pulsatile flow and steady-state flow. They reported that pressure drop across a stenosis remained relatively unchanged (<5%), provided that the mean value of the pulsatile flow rate (time-averaged over a cardiac cycle) equaled the steady state value. Nevertheless, we plan to extend the present work in the future to study the effects of unsteady pulsatile flow. The blood analog fluid used in the *in vitro* model has a Newtonian viscosity of 4.5 cP similar to normal blood viscosity data available in existing literature. The viscosity of blood changes with many factors, and may somewhat impact pressure drop due to variability in viscous losses.

This pilot study is limited by its small sample size. We only validated FFR_3D_ on patients with de novo lesions. Selection bias might be involved. Further, the experiments were conducted with *patient-specific* single coronary vessel models; thus neglecting the effect of branching/bifurcation, serial lesions or collateral flow, which may cause additional levels of pressure drop. Consequently, future studies in a large sample size using *patient-specific aorto-coronary 3D-printed* models that account for the presence of bifurcation and collateral flow should extend our current work.

## Conclusion

In this study, an *in vitro* experimental flow loop using 3D-printed patient-specific coronary arteries was developed. The flow loop essentially modeled physiological coronary circulation, as flow-dependent stenosis resistance in series with a downstream resistance. The main finding of this study was that *3D printed patient-specific* models (FFR_3D_) can be used in a *non-invasive in vitro* environment to quantify coronary artery ischemia with good correlation and concordance to that of invasive FFR.

## Competency In Medical Knowledge

Invasive FFR is the current gold standard not only for evaluating the functional significance of a stenosis but also to guide treatment. In this pilot study, we developed a novel non-invasive diagnostic approach of combining both anatomy and physiology using CCTA scans and 3D printing (FFR_3D_) to evaluate coronary artery ischemia. The FFR evaluated using 3D printed patient-specific models had a good correlation and concordance with invasive FFR.

## Translational Outlook

Future studies in a large sample size using *patient-specific aorto-coronary 3D-printed* models that account for the presence of bifurcation and collateral flow are needed to extend our current work. Furthermore, the technological advancements in deep learning based segmentation approaches, 3D printing methodologies (and materials) and utilization of pressure sensing taps on the 3D printed model for direct pressure measurement could further automate and expedite the entire workflow while testing in a larger cohort.

## Data Availability Statement

The original contributions presented in the study are included in the article/Supplementary Material, further inquiries can be directed to the corresponding author/s.

## Ethics Statement

The studies involving human participants were reviewed and approved by IRB, WCMC. *In vitro* experiments were based on patient's image data and hemodynamic parameters, which were deidentified prior to study. Written informed consent for participation was not required for this study in accordance with the National Legislation and the Institutional Requirements.

## Author Contributions

KK, SD, and BM: designed the study. KK, S-JJ, AZ, AC, SA, AM, PX, and RS: participated in segmenting the image data and 3D printing the *in vitro* models. KK, SA, and AM: *in vitro* experimental setup. KK: analyzed the data and drafted the manuscript with all authors. All authors read and approved the final manuscript.

## Funding

This manuscript was supported, in part, by grants from the National Institutes of Health, the National Heart Lung and Blood Institute (Grant Nos. R01 HL118019, R01 HL115150, and R21 HL132277), as well as from a generous gift from the Dalio Foundation.

## Conflict of Interest

Dr. Min has served on the Scientific Advisory Board of Arineta and GE Healthcare; has received funding from the Dalio Foundation, the National Institutes of Health, and GE Healthcare; and has an equity interest in MDDX and Cleerly. Dr. Shaw receives funding from the National Institutes of Health. KK is an employee at Abbott. However, he was an employee of the Dalio Institute of Cardiovascular Imaging, Weill Cornell Medicine, at the time of both executing the study and writing the manuscript. The remaining authors declare that the research was conducted in the absence of any commercial or financial relationships that could be construed as a potential conflict of interest.

## Publisher's Note

All claims expressed in this article are solely those of the authors and do not necessarily represent those of their affiliated organizations, or those of the publisher, the editors and the reviewers. Any product that may be evaluated in this article, or claim that may be made by its manufacturer, is not guaranteed or endorsed by the publisher.
