# Supplementary material for: Improved Functional Assessment of Ischemic Severity Using 3D Printed Models
Source: Front Cardiovasc Med. 2022 Jun 30;9:909680. doi: 10.3389/fcvm.2022.909680 (PMC9279862; doi:10.3389/fcvm.2022.909680)
Supplement: Supplementary file 1 [file Data_Sheet_1.pdf]

Supplementary information:

The CREDENCE TRIAL INVESTIGATORS

James K. Min, MD, Department of Radiology, New York-Presbyterian Hospital and the Weill Cornell Medical College, New York; Wijnand J. Stuijfzand, MD, Department of Radiology, New York-Presbyterian Hospital and the Weill Cornell Medical College, New York; Alexander R. van Rosendael, MD, Department of Radiology, New York-Presbyterian Hospital and the Weill Cornell Medical College, New York; Fay Y. Lin, MD, Department of Radiology, New York-Presbyterian Hospital and the Weill Cornell Medical College, New York; Hyuk-Jae Chang, MD, PhD, Division of Cardiology, Yonsei University Health System, Seoul, South Korea; Inge J. van den Hoogen, MD, Department of Radiology, New York-Presbyterian Hospital and the Weill Cornell Medical College, New York; Umberto Gianni, MD, Department of Radiology, New York-Presbyterian Hospital and the Weill Cornell Medical College, New York; Jung Hyun Choi, MD, PhD, Pusan National University Hospital, Busan, South Korea; Joon-Hyung Doh, MD, Division of Cardiology, Inje University Ilsan Paik Hospital, Goyang, South Korea; Ae-Young Her, MD, Kang Won National University Hospital, Chuncheon, South Korea; Bon-Kwon Koo, MD, PhD, Department of Internal Medicine, Seoul National University Hospital, Seoul, South Korea; Chang-Wook Nam, MD, PhD, Cardiovascular Center, Keimyung University Dongsan Hospital, Daegu, South Korea; Hyung-Bok Park, MD, Division of Cardiology, Department of Internal Medicine, International St Mary's Hospital, Catholic Kwandong University College of Medicine, Incheon, South Korea; Sang-Hoon Shin, MD, Division of Cardiology, Department of Internal Medicine, Ewha Women’s University Seoul Hospital, Seoul, South Korea; Jason Cole, MD, Mobile

Cardiology Associates, Mobile, Alabama; Alessia Gimelli, MD, Department of Imaging, Fondazione Toscana Gabriele Monasterio, Pisa, Italy; Muhammad Akram Khan, MD, Cardiac Center of Texas, McKinney; Bin Lu, MD, State Key Laboratory of Cardiovascular Disease, Fuwai Hospital, Beijing, China; Yang Gao, MD, State Key Laboratory of Cardiovascular Disease, Fuwai Hospital, Beijing, China; Faisal Nabi, MD, Houston Methodist Hospital, Houston, Texas; Ryo Nakazato, MD, Cardiovascular Center, St. Luke's International Hospital, Tokyo, Japan; U. Joseph Schoepf, MD, Medical University of South Carolina, Charleston; Roel S. Driessen, MD[2]; Michiel J. Bom, MD, Amsterdam University Medical Center, VU University Medical Center, Amsterdam, the Netherlands; Randall Thompson, MD, St Luke's Mid America Heart Institute, Kansas City, Missouri; James J. Jang, MD, Kaiser Permanente Hospital, Oakland, California; Michael Ridner, MD, Heart Center Research, LLC, Huntsville, Alabama; Chris Rowan, MD, Renown Heart and Vascular Institute, Reno, Nevada; Erick Avelar, MD, Oconee Heart and Vascular Center, St Mary's Hospital, Athens, Georgia; Philippe Généreux, MD, Gagnon Cardiovascular Institute at Morristown Medical Center, Morristown, New Jersey; Paul Knaapen, MD, PhD, Amsterdam University Medical Center, VU University Medical Center, Amsterdam, the Netherlands; Guus A. de Waard, MD, Amsterdam University Medical Center, VU University Medical Center, Amsterdam, the Netherlands; Gianluca Pontone, MD, PhD, Centro Cardiologico Monzino, IRCCS Milan, Italy; Daniele Andreini, MD, PhD, Centro Cardiologico Monzino, IRCCS Milan, Italy; Mouaz H. Al-Mallah, MD, Houston Methodist DeBakey Heart and Vascular Center, Houston Methodist Hospital, Houston, Texas; Yao Lu, MSc, Department of Radiology, New York-Presbyterian Hospital and the Weill Cornell Medical College, New York;

Daniel S. Berman, MD, Department of Imaging and Medicine, Cedars Sinai Medical Center, Los Angeles, California; Jagat Narula, MD, PhD, Mount Sinai Heart, Icahn School of Medicine at Mount Sinai, New York, New York; Jeroen J. Bax, MD, PhD, Department of Cardiology, Leiden University Medical Center, Leiden, the Netherlands; Leslee J. Shaw, PhD, Department of Radiology, New York-Presbyterian Hospital and the Weill Cornell Medical College, New York; Dante Chiappino, MD, Department of Imaging, Fondazione Toscana Gabriele Monasterio, Pisa, Italy; Venkata Chilakapati, MD, Ahmed Ladak, MD, Irfan Ullah, MD, Cardiac Center of Texas, McKinney, Texas; Chaowei Mu, MD, State Key Laboratory of Cardiovascular Disease, Fuwai Hospital, Beijing, China; Hiroyuki Niinuma, MD, PhD, Cardiovascular Center, St. Luke's International Hospital, Tokyo, Japan; Daniel H. Steinberg, MD, Medical University of South Carolina Charleston, SC; Roel Driessen, MD, University Medical Center, Amsterdam, Netherlands; Jessica M. Peña, MD, Erica C. Jones, MD, Subhi J. Al'Aref MD, Daria Frestad MD, Amit Kumar MSc, Department of Radiology, New York-Presbyterian Hospital, Weill Cornell Medicine, New York, New York.

This supplementary file describes the determination of hyperemia from the in vitro experiments and thus FFR_{3D}.

Pressure drop- Flow rate Characteristics. Young et al.[1-4] developed fluid dynamic equations that describe the relationship between the pressure distal to a stenosis and the flow. The ΔP-Q characteristics across the stenosis can be expressed as a quadratic relation $\Delta P = P_a - P_d = A_v Q + BQ^2$; where A_v and B are the viscous loss along the stenosis and exit loss (due to the change in momentum) coefficients, respectively. When coronary flow increases, the coronary perfusion pressure distal to the stenosis decreases in a nonlinear fashion, according to the equation, $P_d = P_a - A_v Q - BQ^2$.

Regression analysis was used to fit a quadratic relationship in the (i) pressure drop (ΔP) – flow rate (Q) and (ii) pressure ratio (P_d/P_a) – flow rate (Q) characteristics from the *in vitro* experiment, respectively, as shown in Supplementary Figures 1 and 2. Similarly, as demonstrated in Supplementary Figure 3, linear regression analysis was used to fit a linear curve on the *clinical* hyperemic coronary flow reserve (CFR) – distal pressure (P_d) characteristics.

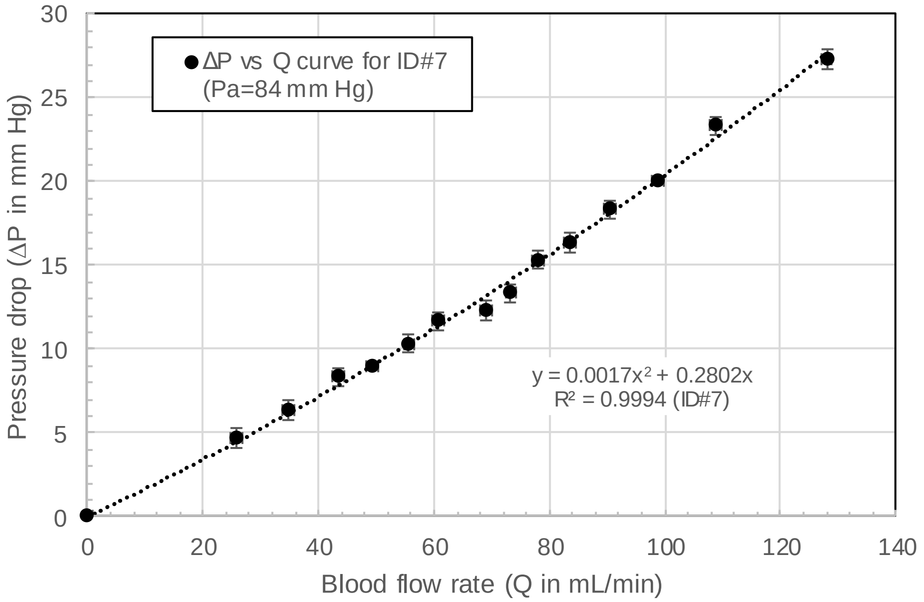

Supplementary Figure 1: Pressure drop (ΔP=Pa−Pd) vs flow (Q) characteristics for a 3D printed patient-specific LAD model at fixed aortic pressures of 84 mm Hg (from ICA). P_a, aortic pressure in mm Hg; P_d, distal pressure in mm Hg; Q, flow rate in mL/min.

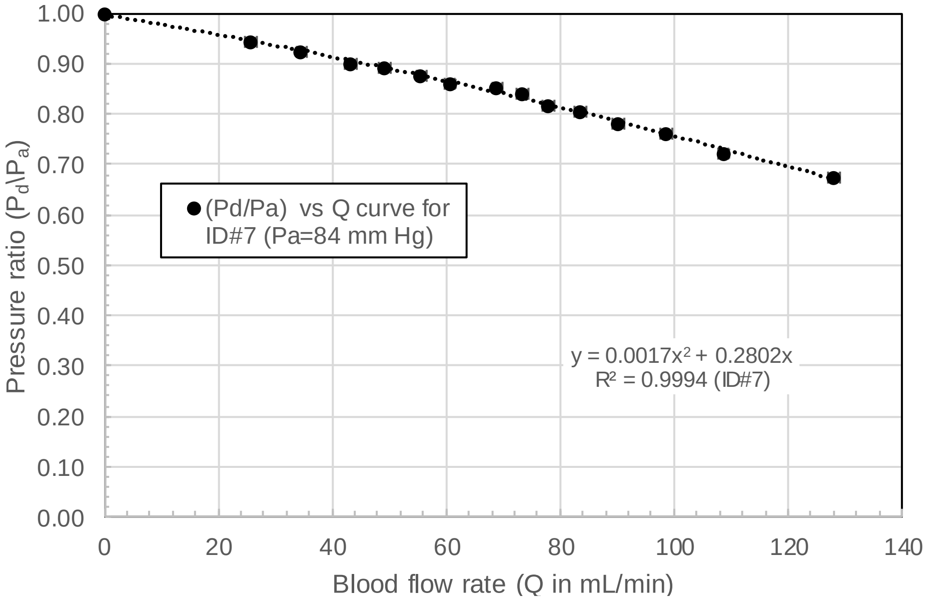

Supplementary Figure 2: Pressure ratio vs flow characteristics for a 3D printed patient-specific LAD model at fixed patient-specific aortic pressures of 84 mm Hg (from ICA). P_a, aortic pressure in mm Hg; P_d, distal pressure in mm Hg; Q, flow rate in mL/min.

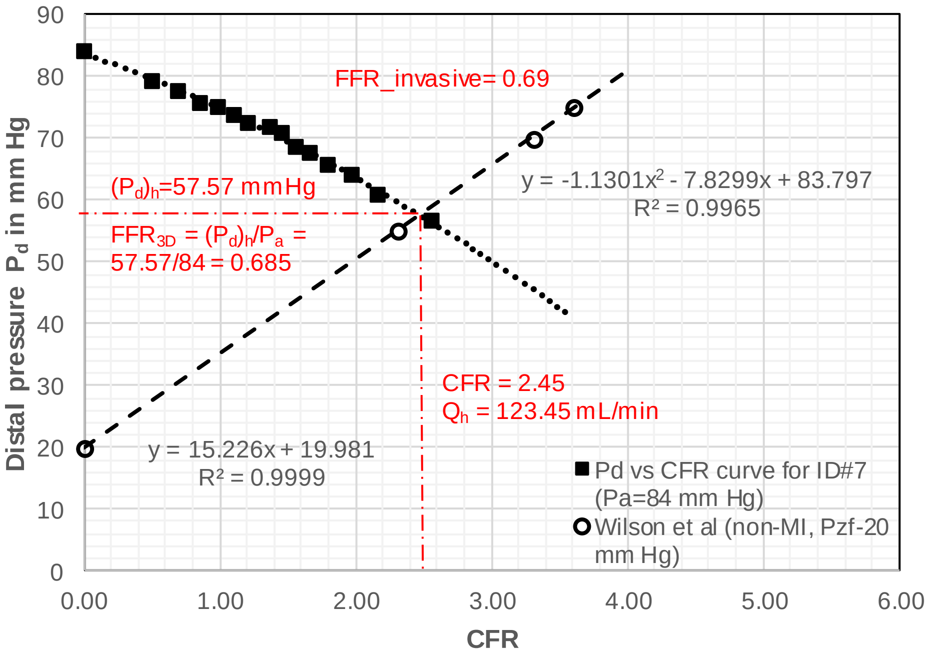

<u>Supplementary Figure 3:</u> Pd vs CFR characteristics for a 3D printed patient-specific LAD model at fixed aortic pressures of 84 mm Hg (from ICA). CFR indicates coronary flow reserve; P_d, pressure distal to stenosis; DS, Pzf, zero-flow mean pressure; Wilson et al[5]

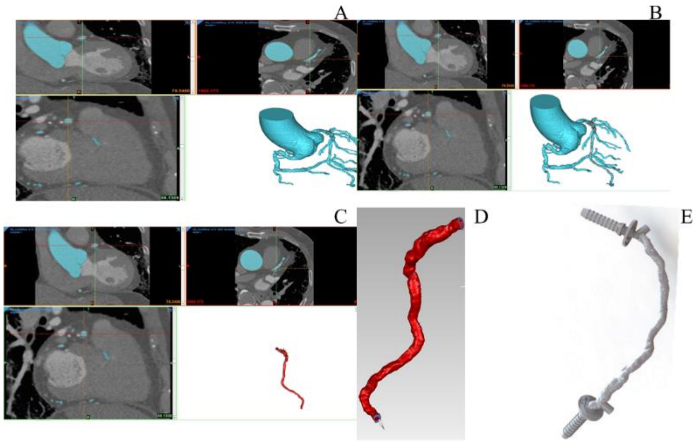

<u>Supplementary Figure 4:</u> Comparison of the aorto-coronary and coronary segmented model with the 3D printed part. (A,B) Segmented aortocoronary model in different views (C) extraction of single coronary artery from aortocoronary model (D) remeshing the coronary mesh and thickening it to 1 mm (representative of coronary thickness) (E) 3D model with barbs attached for maintain a leakless watertight fit in the invitro setup

<u>References:</u>

1. Young DF, Cholvin NR, Kirkeeide RL, Roth AC. Hemodynamics of arterial stenoses at elevated flow rates. Circ Res 1977;**41**(1):99-107.
2. Young DF, Cholvin NR, Roth AC. Pressure drop across artificially induced stenoses in the femoral arteries of dogs. Circ Res 1975;**36**(6):735-43.
3. Young DF, Tsai FY. Flow characteristics in models of arterial stenoses. I. Steady flow. J Biomech 1973;**6**(4):395-410.
4. Young DF, Tsai FY. Flow characteristics in models of arterial stenoses. II. Unsteady flow. J Biomech 1973;**6**(5):547-59.
5. Wilson RF, Johnson MR, Marcus ML, Aylward PE, Skorton DJ, Collins S, White CW. The effect of coronary angioplasty on coronary flow reserve. Circulation 1988;**77**(4):873-85.
